# Effects of adding N_2_-fixing *Rhodopseudomonas palustris* to stimulate the growth and yield of canary melon (*Cucumis melo* L.)

**DOI:** 10.1371/journal.pone.0329938

**Published:** 2025-08-07

**Authors:** Le Minh Tuan, Nguyen Phuong Truc Huyen, Vo Thi Bich Thuy, Le Thanh Quang, Le Thi My Thu, Nguyen Thi Xuan Dao, Tran Chi Nhan, Ly Ngoc Thanh Xuan, Nguyen Quoc Khuong

**Affiliations:** 1 Experimental and Practical Area, An Giang University, Vietnam National University Ho Chi Minh City, An Giang, Vietnam; 2 Faculty of Crop Science, College of Agriculture, Can Tho University, Can Tho, Vietnam; College of Horticulture and Forestry (Dr YS Parmar University of Horticulture and Forestry), Nauni, Solan (HP), INDIA

## Abstract

This study evaluated the effects of a mixture of four N_2_-fixing strains of *Rhodopseudomonas palustris*-VNW64, VNS89, TLS06, and VNS02-(PNSB) on soil properties, nitrogen (N) uptake, plant growth, and yield of canary melon cultivated in alluvial soil. A greenhouse experiment was conducted using a completely randomized block design with eight treatments: (i) 100% N of recommended fertilizer formula (RFF), (ii) 85% N of RFF, (iii) 70% N of RFF, (iv) 100% N of RFF + PNSB, (v) 85% N of RFF + PNSB, (vi) 70% N of RFF + PNSB, (vii) PNSB only, and (viii) no fertilization. The application of PNSB improved soil pH and available N concentrations. The highest N uptake (33.9 kg N ha ⁻ ¹) was recorded in the 100% RFF + PNSB treatment. Notably, the 70% RFF + PNSB treatment achieved comparable N uptake (27.7 kg N ha ⁻ ¹) to the 100% RFF treatment (28.6 kg N ha ⁻ ¹). The 85% RFF + PNSB treatment maintained plant height and yield equivalent to the 100% RFF treatment. These results suggest that supplementing with PNSB can reduce N fertilizer application by up to 15% without compromising crop performance. The PNSB mixture should be further tested under a field trial.

## Introduction

Canary melon (*Cucumis melo* L.) is valued for its high nutritional contents, including ascorbic acid, carotene, folic acid, and potassium, as well as various bioactive compounds [1]. Economically important, its cultivation in greenhouses is expanding globally due to challenges associated with field production [2]. Among agronomic practices, nitrogen (N) fertilization is a major determinant of fruit yield and quality in melon cultivation [3,4]. Nitrogen is essential for plant growth and metabolism [5], yet conventional N fertilizer use often achieves less than 50% uptake efficiency [6], with the remainder lost via volatilization, leaching, or runoff—contributing to air and water pollution [7,8]. Modern crop production greatly depends on the enormous application of N fertilizer, but its efficiency in using is not over 50% [9], which leads to serious air and water contamination. Enhancing N use efficiency is therefore critical for sustainable agriculture [6].

Free-living N_2_-fixing bacteria, such as strains of the purple non-sulfur bacterium *Rhodopseudomonas palustris*, can convert atmospheric N₂ into plant-available NH₄⁺ and have a great potential in reducing the N fertilizer used [10]. Shuang et al. [11] and Hsu et al. [12] claim that inoculation with *R. palustris* has enhanced not only the availability of nutrients in soil but also nutrients uptake of cucumber and Chinese Pakchoi cabbage. Recently, the N_2_-fixing strains of *R. palustris* VNW64, VNS89, TLS06 and VNS02 have been selected [13,14] for their robust performance in rice, enhancing both growth and yield in saline and acid-sulfate soils [15]. Recently, three PNSB strains, *Luteovulum sphaeroides* EPS18, EPS37, and EPS54, have been applied to improve rice tolerance against salt stress [16]. Moreover, These strains also possess phosphorus-solubilizing activity and can thrive under both aerobic and anaerobic conditions [17–19], such as four PNSB isolated from saline acid sulfate soil [20].

Therefore, these strains of *R. palustris* are promising in decreasing the N fertilizer used for terrestrial plants, such as canary melon. Given these multi-functional traits, we hypothesized that a mixture of the four *R. palustris* strains could reduce the requirement for chemical N fertilizer while maintaining or improving the growth and yield of canary melon in alluvial soil. Accordingly, this study aimed to evaluate the effects of mixed *R. palustris* inoculation on soil fertility, plant N uptake, growth parameters, and fruit yield of canary melon cultivated under greenhouse conditions.

## Materials and methods

### Materials

The experiment was carried out at the greenhouse of the Agricultural Research and Experimental Farm, College of Agriculture, Can Tho University, from December 2020 to February 2021.

The characteristics of alluvial soil used for cultivating canary melon were described in Table 1.

**Table 1:** Initial characteristics of soil used for canary melon cultivation.

Property	Unit	Depth (cm)	
		0-20	20-40
**pH**_**H2O**_	–	4.39 ± 0.70	3.84 ± 0.50
**pH**_**KCl**_	–	3.40 ± 0.33	2.71 ± 0.01
**EC**	mS cm^-1^	0.495 ± 0.191	0.320 ± 0.028
**OM**	% C	6.28 ± 0.14	7.78 ± 0.56
**Total N**	% N	0.161 ± 0.001	0.102 ± 0.015
**NO**_**3**_^**-**^	mg NO_3_^-^ kg_soil_^-1^	8.97 ± 0.70	9.52 ± 0.47
**NH**_**4**_^**+**^	mg NH_4_^ +^ kg_soil_^-1^	7.28 ± 0.58	8.38 ± 0.58
**Total P**	% P_2_O_5_	0.068 ± 0.019	0.044 ± 0.0003
**Soluble P**	mg P kg_soil_^-1^	56.3 ± 4.70	51.3 ± 0.97
**Al-P**	mg P kg_soil_^-1^	52.5 ± 5.48	34.3 ± 3.03
**Fe-P**	mg P kg_soil_^-1^	375.5 ± 10.5	238.2 ± 2.53
**Ca-P**	mg P kg_soil_^-1^	20.2 ± 3.43	17.8 ± 3.43
**CEC**	meq 100 g_soil_^-1^	13.7 ± 1.70	13.4 ± 2.72
**Na** ^**+**^	meq 100 Na^ +^ g_soil_^-1^	0.377 ± 0.038	0.283 ± 0.121
**K** ^**+**^	meq 100 K^ + ^g_soil_^-1^	0.393 ± 0.039	0.407 ± 0.006
**Mg** ^**2+**^	meq 100 Mg^2 +^ g_soil_^-1^	1.85 ± 0.18	1.65 ± 0.29
**Ca** ^**2+**^	meq 100 Ca^2 + ^g_soil_^-1^	1.54 ± 0.24	1.08 ± 0.24

Values are mean of four replicates, ± standard deviation, EC: Electrical conductivity, OM: Organic matter, CEC: Cation exchangeable capacity.

The canary melon cultivar (F1 hybrid) used in this study is known for its firm, smooth, and crunchy flesh with a carbohydrate content of 15–18%. It grows optimally at temperatures ranging from 20–32 °C, with a growth cycle of 60–65 days.

The BIM medium used for bacterial culture consisted of the following components: 1.0 g (NH_4_)_2_SO_4_, 0.5 g K_2_HPO_4_, 0.2 g MgSO_4_, 2.0 g NaCl, 5.0 g NaHCO_3_, 1.5 g yeast extract, 1.5 g glycerol, 0.03 g L-cysteine and 1.5% agar for 1 L of distilled water [21].

The strains of *Rhodopseudomonas palustris* VNW64, VNS89, TLS06 and VNS02 were all able to fix N_2_ from the air [13,14]. In addition, these strains were also capable of solubilizing phosphorus (P), and producing 5-aminolevulinic acid (ALA), exopolysaccharides (EPS), and siderophores [14].

The fertilizers used were made of NPK 1(6-16-8), KCl, organic fertilizers and super phosphate.

### Methods

Experimental design: The experiment followed a completely randomized design with 8 treatments fertilized: (i) with 100% N of RFF (the control treatment), (ii) with 85% N of RFF, (iii) with 70% N of RFF, (iv) with 100% N of RFF plus the four PNSB strains, (v) with 85% N of RFF plus the four PNSB strains, (vi) with 70% N of RFF plus the four PNSB strains, (vii) with only the four PNSB strains, (viii) with no chemical fertilizer and no bacteria. Each treatment had 3 replicates, each of which were 5 m^2^ in each plot in the greenhouse.

The culture media were prepared according to a description of Brown [21] and modified by Khuong et al. [13]. Each strain was raised separately in BIM media (pH 4.5) under microaerobic light conditions for 48 h. Then, the bacterial broth was centrifuged at 6,000 rpm for 15 min, from which the bacterial cells were collected and rinsed twice with peptone 0.1%. The bacterial suspension was adjusted to reach an OD_660_ of 0.8 in a spectrophotometer to obtain a bacterial density of 10^8^ cells mL^-1^. The bacterial mixture used was a combination of the four bacterial strains at the same volume.

Inoculating the bacteria to seeds: Seeds of canary melon were soaked in warm water (1:1 ratio) for an hour, before being incubated for germination. Then, the germinated seeds (200 seeds) were divided into 2 equal portions, one of which was transferred into a 100 mL beaker containing the four-strain mixture of *R. palustris* VNW64, VNS89, TLS06, and VNS02, while the other half was put into a 100 mL beaker of distilled water for an hour. Then, these seeds from both halves were sown on each sowing tray. When there was a true leaf (10 days after sowing, DAS), pesticides were applied. The mixture of *Rhodopseudomonas* spp. was reapplied at 4 stages, including 10 DAS, and 5, 25, and 40 days after plantation, at density of 1 x 10^9^ CFU g^-1^ of dry soil weight.

### Soil analysis procedure

The analytic methods for soil characteristics were described by Sparks et al. [22] as follows: for the pH and the EC, the pH_H2O_ pH_KCl_ values were extracted by deionized water and KCl 1 M at a ratio of 1:5 of soil to deionized water or 1 M KCl. Then, the solvents were used to measure the pH and the EC by a pH meter and an EC meter in the solvent extracted by deionized water. The Kjeldahl method was used to determine the total N content, i.e., samples were turned into inorganic compounds by saturated H_2_SO_4_ and CuSO_4_-Se, then the N in inorganic form was titrated with H_2_SO_4_ 0.01 N. NH_4_^+^ and NO_3_^-^ concentrations were extracted by KCl 2 M and their concentrations were measured by blue phenol method and colorimetrically quantified by a mixture of HCl 0.5 M, vanadium (III) chloride and sulfanilamide at a wavelength of 650 nm for NH_4_^+^ and 540 nm for NO_3_^-^_,_ respectively. Total P content was determined by the inorganic P converted from organic P by saturated H_2_SO_4_ and HClO_4_. The inorganic P was indicated in colors by a mixture of sulfuric acid, ammonium molybdate, acid ascorbic and antimony ammonium tartrate at a wavelength of 880 nm. Soluble P was determined by Bray II method with a ratio of 1 soil: 7 NH_4_F 1 M + HCl 0.5 M mixture. The insoluble compounds, including Al-P, Fe-P and Ca-P, were extracted by NH_4_F 0.5 M, NaOH 0.1 M and H_2_SO_4_ 0.25 M, respectively then revealed in colors by H_2_SO_4_ 2.5 M, ammonium molybdate, ascorbic acid and potassium antimonyl tartrate at a wavelength of 880 nm. CEC was extracted by BaCl_2_ 0.1 M, and the solution from that was used to determine the concentrations of K^+^, Na^+^, Ca^2+^ and Mg^2+^ by a spectrophotometer at a wavelength of 766, 589, 422.7 and 285.2 nm, respectively.

### Plant samples analysis procedure

The content of nitrogen in stem, leaves and fruits was determined according to a method of Houba et al. [23]. In brief, stem, leaves and fruits samples in every treatments at harvest oven-dried up at 70 °C for 72 h. After that, the dry samples were smashed via a sieve of 0.5 mm and turned into inorganic forms by saturated H_2_SO_4_ and salicylic acid in order to convert the organic N to inorganic N. The inorganic solution was applied for N concentration determination by Kjeldahl method.

Dry biomass (kg plant^-1^): all of the stem, leaves, and fruits of were weighted and their weight was converted into t ha^-1^.

Total nitrogen uptake by the plant was calculated by multiplying shoot dry weight by its N content. Fruit yield per hectare was extrapolated from the fruit fresh weight harvested per plot.

### Agronomic traits

In each plot, 6 plants were chosen to evaluate the agronomic characteristics at 38 DAP, which was described as below:

• Plant height (cm): was measured from the ground above 2 cotyledons to the apical meristem of a plant.
• Number of leaves (leaves): was counted from the first true leaf (rough leaf) to the peak leaf, which was bigger than 2 cm.
• Stem diameter (cm) was measured at a position 2 cm from the ground.
• Fruit-bearing leaves (leaves): were counted from the first true leaf to the leaf that bears a fruit.

### Yield components

The fruits of canary melon were harvested at 64 DAP, and 6 fruits in each plot were randomly collected (avoiding the fruits at both ends of the row) to evaluate the yield component and the quality, and to analyze the nutrient components:

- Fruit height (cm) was measured as the length from both ends of a fruit.
- Fruit perimeter (cm) was measured at the middle of a fruit.
- Fruit weight (kg fruit^-1^): was the mean weight from weight of the 6 collected fruits.

### Fruit yield

- Observed yield (t ha^-1^): all of the fruits of a plot were weighed. Then, the yield was converted into t ha^-1^.

### Fruit quality

- Hardness: 1-cm-thick flesh parts at 3 positions were cut out by a blade and used to measure fruit hardness by Fruit Pressure Tastar-FT 327, then an average value was derived from that.
- Color: colors at the top, middle, and bottom of a fruit were measured by Colorimeter CR-200 to infer index of L*, a*, and b*.
- Fruit shell thickness: a canary melon was cut vertically, and its fruit shell thickness was measured at the upper, middle, and lower of it, which were calculated for a mean. The upper and lower parts were 2 cm from the top and bottom of the fruit.
- Fruit flesh thickness: in the same line as above, a canary melon was cut vertically, and its fruit flesh thickness was an average value from the upper, middle, and lower parts of it. The upper and lower parts were 2 cm from the top and bottom of the fruit
- Concentration of NO_3_^-^: 3 g sample was smashed with K_2_SO_4_ 0.05% and heated for 30 min. Then, it was allowed to cool and adjusted to 50 mL with K_2_SO_4_ 0.05%, and filtered with filter paper. 5 mL of the filtrated sample was dried out and added with 1 mL disulfonic acid and 25 mL distilled water, the pH was adjusted to 7.0 with NaOH 0.1 M, and the volume was adjusted to 50 mL. Finally, the mixture was measured by a spectrophotometer at 436 nm.
- Brix index of the fruit flesh (%): the flesh at the upper, middle, and lower parts of a fruit were collected with a blade and the derived fruit juice. The juice was then dropped directly onto the prism of a refractometer (Atago ATC-1). From that, the Brix value could be read.
- Vitamin C: 5 g of the fruit sample was smashed with 20 mL HCl 1%. Then, oxalic acid 1% was used to adjust the volume to 100 mL, and the mixture was shaken steadily and filtrated by filter paper. 10 mL of the filtrated solution was titrated with 2,6 Dichlorophenol indophenol 0.001 N.
- Total acid: 2 g of sample was smashed with 50 mL distilled water. 2 mL of the solution was centrifuged at 3,000 rpm for 3 min. 1 mL of the sample solution and 9 mL of distilled water was titrated by phenolphthalein 1% and NaOH 0.01 N.
- Storing duration: fruits were left at room temperature and daily checked until corruption appeared, which was a kind of rotten smell.

### Statistical analysis

Statistical analyses were performed using SPSS software (version 20.0). Data were analyzed using one-way analysis of variance (ANOVA). Treatment means were compared using Duncan’s multiple range test at a significance level of p ≤ 0.05.

## Results

### The influence of N_2_-fixing purple non-sulfur bacteria on alluvial soil fertility

pH_H2O_ and pH_KCl_ values among treatments differed from each other at 5% significance. At N fertilizer levels of 70–100% N of RFF, in the treatments fertilized with the mixture of the four PNSB strains, pH_H2O_ and pH_KCl_ were approximately 5.00–5.46 and 4.29–4.90, higher than 4.38–4.44 and 3.86–4.07 in the treatments fertilized with no bacteria. Additionally, in the treatment fertilized with only the four PNSB strains, pH_H2O_ (5.40) and pH_KCl_ (4.98) were higher than those in the treatment fertilized with no bacteria, where the pH_H2O_ and pH_KCl_ were 5.15 and 4.18, respectively (Table 2). For EC value, at the same N fertilizer level, in the treatments supplemented with the four PNSB strains, EC values were lower than those in the treatments fertilized with only chemical fertilizer, 1.05–1.27 mS cm^-1^ compared to 1.43–1.58 mS cm^-1^. However, in the absence of chemical fertilizer, the treatment fertilized with the supplementation of the four PNSB strains and the one without had the equivalent EC values in depth of 0–20 cm (Table 2).

**Table 2:** Influences of N_2_-fixing purple nonsulfur bacteria *R. palustris* on alluvial soil fertility in depth of 0-20 cm.

Treatment	pH_H2O_	pH_KCl_	EC	Total N	NH_4_^+^	NO_3_^-^	Total P	Soluble P	Insoluble P forms			CEC	Cations			
									Al-P	Fe-P	Ca-P		K^+^	Na^+^	Ca^2+^	Mg^2+^
	–	–	(mS cm^-1^)	(%)	(mg kg_soil_^-1^)		(%)	(mg kg_soil_^-1^)				(meq 100 g_soil_^-1^)				
100% N	4.44 ± 0.16^c^	3.98 ± 0.18^c^	1.58 ± 0.02^a^	0.124 ± 0.02	16.1 ± 1.8^b^	96.6 ± 11.8^a^	0.076 ± 0.012	78.2 ± 1.5^bc^	125.2 ± 12.0^a^	450.2 ± 47.0^a^	95.6 ± 2.5^a^	13.1 ± 0.7	0.435 ± 0.034 cd	0.431 ± 0.024^a^	9.19 ± 1.11^bc^	2.21 ± 0.18
85% N	4.38 ± 0.09^c^	3.86 ± 0.04^c^	1.43 ± 0.02^ab^	0.114 ± 0.04	15.3 ± 1.4^b^	104.0 ± 39.4^a^	0.073 ± 0.010	69.1 ± 4.6^de^	127.7 ± 15.3^a^	436.5 ± 0.2^ab^	84.2 ± 3.6^b^	12.9 ± 0.6	0.449 ± 0.001^c^	0.361 ± 0.072^abc^	8.71 ± 0.08^bc^	2.38 ± 0.03
70% N	4.40 ± 0.13^c^	4.07 ± 0.52^c^	1.53 ± 0.16^a^	0.145 ± 0.01	12.0 ± 0.6^c^	103.9 ± 51.7^a^	0.078 ± 0.006	66.0 ± 2.0^e^	115.7 ± 3.3^a^	392.0 ± 9.8^b^	74.3 ± 2.5^c^	13.6 ± 0.3	0.436 ± 0.055 cd	0.359 ± 0.089^abc^	8.91 ± 0.17^bc^	2.02 ± 0.11
100% N + PNSB	5.01 ± 0.03^b^	4.72 ± 0.06^b^	1.18 ± 0.14^c^	0.119 ± 0.01	18.9 ± 0.9^a^	102.5 ± 21.4^a^	0.081 ± 0.001	92.3 ± 4.9^a^	100.7 ± 1.9^b^	324.1 ± 51.8^c^	79.9 ± 2.2^b^	13.7 ± 0.2	0.530 ± 0.043^ab^	0.259 ± 0.044^d^	8.43 ± 0.09^bc^	2.12 ± 0.17
85% N + PNSB	5.46 ± 0.08^a^	4.90 ± 0.33^a^	1.05 ± 0.15^c^	0.121 ± 0.02	18.1 ± 1.4^a^	100.2 ± 3.5^a^	0.075 ± 0.013	82.8 ± 3.0^b^	96.5 ± 0.9^b^	334.3 ± 3.5^c^	79.8 ± 0.5^b^	14.0 ± 0.3	0.549 ± 0.013^a^	0.265 ± 0.022 cd	8.23 ± 0.38^c^	2.29 ± 0.26
70% N + PNSB	5.00 ± 0.05^b^	4.29 ± 0.16^bc^	1.27 ± 0.22^bc^	0.147 ± 0.01	14.3 ± 1.2^b^	97.4 ± 7.0^a^	0.079 ± 0.017	69.4 ± 5.0^de^	94.7 ± 3.5^b^	319.7 ± 22.5 cd	71.6 ± 3.1 cd	13.6 ± 1.6	0.585 ± 0.075^a^	0.350 ± 0.027^abcd^	8.51 ± 0.30^bc^	2.04 ± 0.07
0% N + PNSB	5.40 ± 0.21^a^	4.98 ± 0.44^a^	0.48 ± 0.09^d^	0.131 ± 0.01	11.7 ± 0.6^c^	14.7 ± 2.2^b^	0.069 ± 0.009	74.1 ± 1.6 cd	62.0 ± 2.5^c^	270.4 ± 2/1^d^	59.7 ± 3.3^e^	13.5 ± 0.2	0.456 ± 0.043^bc^	0.314 ± 0.073^bcd^	9.35 ± 0.41^b^	2.13 ± 0.20
0% N	5.15 ± 0.02^b^	4.18 ± 0.34^bc^	0.57 ± 0.11^d^	0.138 ± 0.02	6.87 ± 0.6^d^	16.3 ± 1.5^b^	0.070 ± 0.005	69.5 ± 2.5^de^	96.7 ± 5.8^b^	317.2 ± 3.0 cd	68.4 ± 2.9^d^	13.7 ± 1.7	0.358 ± 0.023^d^	0.366 ± 0.018^ab^	10.75 ± 0.25^a^	2.19 ± 0.17
Significant differences	*	*	*	ns	*	*	ns	*	*	*	*	ns	*	*	*	ns
CV (%)	2.33	6.98	11.5	15.6	7.99	33.0	13.7	4.57	7.27	7.38	3.53	6.62	9.42	16.2	5.17	7.56

Numbers in each column with the same following letters are not significantly different from each other. ns: not significant difference; *: significant difference at 5% according to Duncan’s test; PNSB: Mixture of N_2_-fixing bacteria strains as *R. palustris* VNW64, VNS89, TLS06, and VNS02, N: Nitrogen, P: Phosphorus; EC: Electrical conductivity, CEC: Cation exchangeable capacity.

Concentrations of total N, total P and cation exchangeable capacity and Mg^2+^ changed insignificantly among treatments, i.e., the values fluctuated approximately 0.114–0.147%, 0.069–0.081% and 2.02–2.38 meq 100 g^-1^, and 12.9–14.0 meq Mg^2+^ 100 g^-1^, in depth of 0–20 cm, respectively (Table 2).

**Fig 1:**
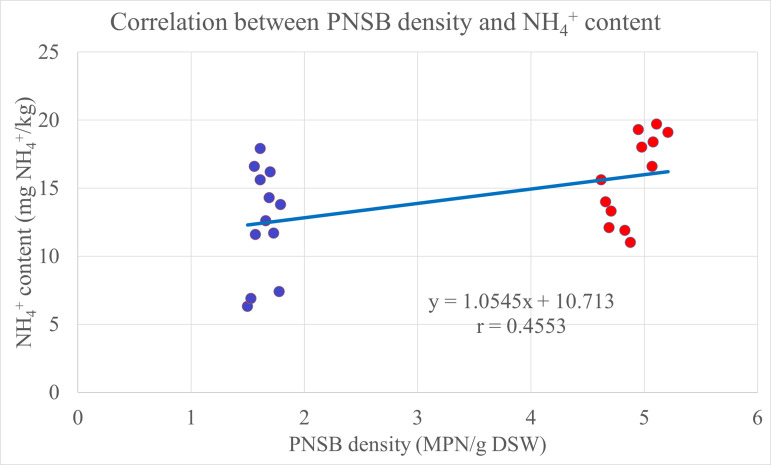
The correlation between the PNSB density and the NH_4_^+^ content in the soil. Blue spots: treatments without PNSB; red spots: treatments with PNSB. DSW: dry soil weight.

The result in Table 2 indicated significant differences at 5% in concentrations of the NH_4_^+^ and NO_3_^-^ among treatments. In detail, at 0% N of RFF, in the treatment supplemented with the four PNSB strains, NH_4_^+^ concentration was 11.7 mg NH_4_^+^ kg^-1^, higher than that in the treatment fertilized with no bacteria, 6.87 mg NH_4_^+^ kg^-1^. In the treatments fertilized with only 85% or only 100% N of RFF, NH_4_^+^ concentrations were equivalent (15.3 and 16.1 mg NH_4_^+^ kg^-1^) and higher than 12.0 mg NH_4_^+^ kg^-1^ in the treatment fertilized with only 70% N of RFF. NH_4_^+^ concentrations in the treatments fertilized with either 70%, 85%, or 100% N of RFF plus the mixture of the four PNSB strains were 14.3, 18.1, and 18.9 mg NH_4_^+^ kg^-1^, higher than those in the treatments that used only chemical fertilizer at the same N fertilizer level. In particular, in the treatment fertilized with 70% N of RFF plus the four PNSB strains, the content of NH_4_^+^ was statistically equal to that in the treatment fertilized with 100% N of RFF. Regarding NO_3_^-^ concentration, treatments fertilized with chemical N fertilizer plus the four PNSB strains were not significantly different from those fertilized with only chemical fertilizer in the content of NO_3_^-^ whose average values were 100.0 and 101.6 mg NO_3_^-^ kg^-1^, respectively. In the same line, in the treatment fertilized with only the four PNSB strains, the content of NO_3_^-^ was 14.7 mg NO_3_^-^ kg^-1^, similar to 16.3 mg NO_3_^-^ kg^-1^ in the treatment fertilized with no fertilizer and no bacteria in the depth of 0–20 cm (Table 2). The PNSB density proportionally correlated with the concentration of NH_4_^+^ in the soil (r = 0.4553) (Fig 1).

Concentrations of soluble P and insoluble ones, including Al-P, Fe-P, and Ca-P, among treatments varied at 5% significance. In detail, soluble P concentration fluctuated approximately 66.0–92.3 mg P kg^-1^, where the highest content was in the treatment fertilized with 100% N of RFF plus the four PNSB strains (Table 2). For Al-P concentration, in the treatments supplemented with the four PNSB strains, the values were 94.7, 96.5 and 100,7 mg P kg^-1^, lower than 115.7, 127.7 and 125.2 mg P kg^-1^ in the treatment fertilized with no bacteria, according to the N fertilizer levels of 70%, 85% and 100% N, respectively. For the content of Fe-P, in the treatments fertilized with only N fertilizer, the values were 392.0, 436.5 and 450.2 mg P kg^-1^, which were significantly dominant in comparison to those in the treatments supplied with the four PNSB strains (319.7, 334.3 and 324.1 mg P kg^-1^) at the same N fertilizer levels of 70%, 85% and 100% N. In the same line, the Ca-P concentrations were 74.3, 84.2 and 95.6 mg P kg^-1^ in the treatments fertilized with only N fertilizer and those in the treatments supplemented with the four PNSB strains were 71.6, 79.8 and 79.9 mg P kg^-1^, following the N fertilizer levels of 70%, 85% and 100% N, respectively. However, at the N fertilizer levels of 70 and 85% N, the Ca-P concentration was equivalent in both treatments fertilized with and without the bacteria. In addition, in the treatment fertilized with only the four PNSB strains, the content of Al-P and Ca-P was all lower than that in the treatment with no fertilization in the depth of 0–20 cm (Table 2).

Table 2 also showed that the concentrations of K^+^, Na^+^ and Ca^2+^ were different from each other among treatments at 5% significance. To be more specific, K^+^ content in the treatments fertilized with only either 70%, 85% or 100% N was lower than that in the treatments supplemented with the four PNSB strains at the same N fertilizer levels, the concentrations were, respectively, 0.436 < 0.585 meq K^+^ 100 g^-1^, 0.449 < 0.549 meq K^+^ 100 g^-1^ and 0.435 < 0.530 meq K^+^ 100 g^-1^. With no chemical fertilizer applied, in the treatment supplemented with only the four PNSB strains, the concentration of K^+^ was 0.456 meq K^+^ 100 g^-1^, higher than that in the treatment fertilized with no bacteria (0.358 meq K^+^ 100 g^-1^). In the same trend, the Na^+^ concentrations dramatically ranged from 0.259 to 0.431 meq Na^+^ 100 g^-1^ among treatments (Table 2). For the content of Ca^2+^, in the treatment applied with no bacteria and no chemical fertilizer as well, the result was 10.7 meq Ca^2+^ 100 g^-1^, higher than that in the treatment supplemented with the four PNSB strains (9.35 meq Ca^2+^ 100 g^-1^). In the treatment fertilized with N fertilizer plus the four PNSB strains and in the one fertilized with only N fertilizer, Ca^2+^ concentrations were, on average, 8.39 and 8.94 meq Ca^2+^ 100 g^-1^ in the depth of 0–20 cm (Table 2).

Furthermore, the soil properties at a depth of 20–40 cm were also recorded in S1 Table.

### The influences of N_2_-fixing purple non-sulfur bacteria on N uptake in canary melon

From the Table 3, N concentrations in stem, leaves and fruits were influenced significantly at 5% among treatments. For the N concentrations in stem, leaves, without chemical fertilizer, in the treatment supplemented with the four PNSB, the content was 2.42%, higher than 1.91% in the one applied without bacteria. The N concentrations in the treatments fertilized with N fertilizer plus the four PNSB strains were insignificantly different from each other, ranging from 2.52 to 2.62%, but higher than those in the treatments used only chemical fertilizer at the same N fertilizer levels (2.27–2.44%). For the N concentrations in fruits, at 100% N of RFF, the outcome was 0.791%, higher than 0.720% and 0.700% at 85% and 70% N of RFF, respectively. In the treatment supplemented with only the four PNSB strains, the N concentration in fruits was 0.669%, higher than that in the treatment applied with no bacteria and no chemical fertilizer (0.543%). On the other hand, in the treatments fertilized with both chemical fertilizer N levels and the four PNSB strains, the N concentration ranged from 0.747% to 0.829%. Interestingly, the treatment fertilized with 85% N of RFF plus the four PNSB strains was equivalent to the one fertilized with only 100% N of RFF in the N concentration (Table 3).

**Table 3:** Influences of N_2_-fixing purple nonsulfur bacteria *R. palustris* on N concentration, biomass and N uptake in canary melon cultivated on alluvial soil.

Treatment	N concentration (%)		Biomass (kg ha^-1^)		N uptake (kg ha^-1^)		Total N uptake (kg ha^-1^)
	Stem, leaves	Fruits	Stem, leaves	Fruits	Stem, leaves	Fruits	
**100% N**	2.44 ± 0.10^bc^	0.791 ± 0.003^ab^	628.6 ± 6.2^b^	1674.9 ± 37.4^b^	15.4 ± 0.7^b^	13.2 ± 0.3^b^	28.6 ± 0.7^c^
**85% N**	2.34 ± 0.13 cd	0.720 ± 0.024^c^	619.9 ± 11.4^bc^	1546.0 ± 54.8^c^	14.5 ± 0.9^bc^	11.1 ± 0.7^c^	25.6 ± 1.1^d^
**70% N**	2.27 ± 0.14^d^	0.700 ± 0.010 cd	580.9 ± 10.1^c^	1462.4 ± 30.0^d^	13.2 ± 1.0^c^	10.2 ± 0.3^d^	23.4 ± 0.8^e^
**100% N + PNSB**	2.62 ± 0.06^a^	0.829 ± 0.019^a^	729.0 ± 43.6^a^	1785.3 ± 10.5^a^	19.1 ± 1.3^a^	14.8 ± 0.3^a^	33.9 ± 1.6^a^
**85% N + PNSB**	2.61 ± 0.09^a^	0.791 ± 0.027^ab^	705.0 ± 19.5^a^	1681.7 ± 45.0^b^	18.3 ± 1.1^a^	13.3 ± 0.4^b^	31.7 ± 1.4^b^
**70% N + PNSB**	2.52 ± 0.05^ab^	0.747 ± 0.047^bc^	634.0 ± 20.3^b^	1574.8 ± 71.9^c^	16.0 ± 0.8^b^	11.7 ± 0.7^c^	27.7 ± 0.8^c^
**0% N + PNSB**	2.42 ± 0.04^bcd^	0.669 ± 0.014^d^	605.3 ± 15.9^bc^	1510.1 ± 38.3 cd	14.6 ± 0.5^bc^	10.1 ± 0.1^d^	24.7 ± 0.5^de^
**0% N**	1.91 ± 0.02^e^	0.543 ± 0.044^e^	313.5 ± 44.7^d^	1208.3 ± 15.6^e^	6.03 ± 0.9^d^	6.53 ± 0.6^e^	12.6 ± 0.3^f^
Significant differences	*	*	*	*	*	*	*
CV (%)	3.50	4.37	4.16	2.46	6.01	4.29	3.55

Numbers in each column with the same following letters are not significantly different from each other. *: significant difference at 5% according to Duncan’s test; PNSB: Mixture of N_2_-fixing bacteria strains as *R. palustris* VNW64, VNS89, TLS06 and VNS02, N: Nitrogen.

Dry biomass values in stem, leaves and fruits of canary melon among treatments varied significantly at 5% significance. In the treatments fertilized with only N fertilizer, the biomass values in dry stem, leaves were 580.9–628.6 kg ha^-1^ and these in dry fruits were 1674.9 > 1546.0 > 1462.4 kg ha^-1^, correspondent to the N fertilizer levels of 100%, 85% and 70% N of RFF. In the treatments fertilized with both the N fertilizer levels and the four PNSB strains, the biomass values in dry stem, leaves were 729.0 and 705.0 kg ha^-1^ at 100% and 85% N of RFF, respectively, higher than that at 70% N of RFF, 634.0 kg ha^-1^; in dry fruits, with the supplementation of the four PNSB strains, there were remarkably differences among treatments, and the highest biomass value was 1785.3 kg ha^-1^ at 100% N of RFF, following, 1681.7 kg ha^-1^ was the second highest and at 85% N of RFF, and the lowest one was 1574.8 kg ha^-1^ at 70% N of RFF. Notably, in the treatment fertilized with 85% N of RFF plus the four PNSB strains, the biomass value in dry stem, leaves was 634.0 kg ha^-1^, equivalent to that in the treatment fertilized with only 100% N of RFF (628.6 kg ha^-1^). In the same line, for the biomass in dry fruits, the treatment fertilized with 85% N of RFF plus the four PNSB strains (1681.7 kg ha^-1^) was statistically the same to the treatment fertilized with only 100% N of RFF (1674.9 kg ha^-1^). Additionally, in the treatment supplemented with only the four PNSB strains, the biomass values were 605.3 kg ha^-1^ in dry stem leaves and 1510.5 kg ha^-1^ in dry fruits, higher than those in the treatment supplemented with no bacteria and no chemical fertilizer (313.5 and 1208.3 kg ha^-1^, respectively) (Table 3).

**Fig 2:**
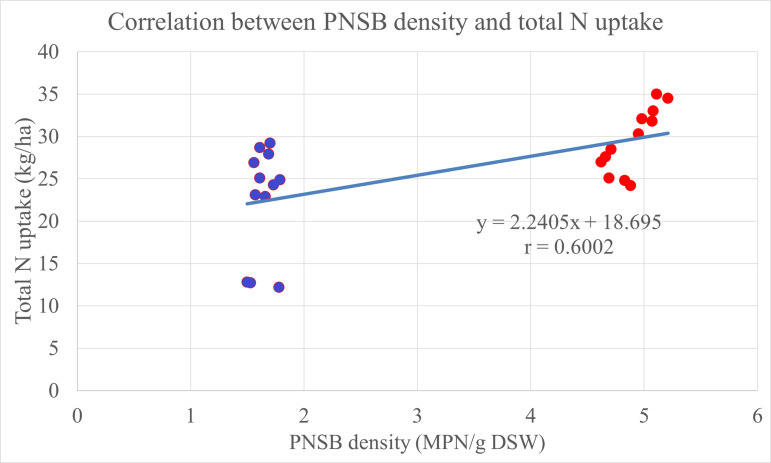
The correlation between the PNSB density in the soil and the total N uptake in the plant. Blue spots: treatments without PNSB; red spots: treatments with PNSB. DSW: dry soil weight.

The result in Table 3 indicated that N uptake in stem, leaves and fruits and total N uptake had differences among treatments at 5% significance. In case of no chemical fertilizer applied, in the treatment supplemented with the four PNSB strains, N uptake in stem, leaves and fruits and total N uptake outweighed those in the treatment supplemented with no bacteria. For N uptake in stem, leaves, in the treatment fertilized with 100% N of RFF, uptake was 15.4 kg N ha^-1^ and higher than that in the treatment fertilized with 70% N of RFF (13.2 kg N ha^-1^). In the same line, in the treatments fertilized with 100% and 85% N of RFF plus the four PNSB, the N uptake in dry stem leaves were equivalently 19.1 and 18.3 kg N ha^-1^, respectively, and all higher than 16.0 kg N ha^-1^ in the treatment fertilized with 70% N of RFF plus the four PNSB strains. Noticeably, the treatment fertilized with 70% N of RFF plus the four PNSB strains had an equivalent N uptake in stem, leaves to the treatment fertilized with only 100% N of RFF. The N uptake in stem, leaves in the treatment fertilized with only the four PNSB strains was 14.6 kg N ha^-1^ and higher than the treatment with no fertilization (6.03 kg N ha^-1^). Along with the N fertilizer levels of 0, 70, 85, 100% N of RFF, the N uptake in fruits were 6.53 < 10.2 < 11.1 < 13.2 kg N ha^-1^ and total N uptake was 12.6 < 23.4 < 25.6 < 28.6 kg N ha^-1^ in the case without the four PNSB strains, and in the case with the four PNSB strains, N uptake was 10.1 < 11.7 < 13.3 < 14.8 kg N ha^-1^ and 24.7 < 27.7 < 31.7 < 33.9 kg N ha^-1^, respectively. Furthermore, N uptake in fruits and total N uptake in the treatments fertilized with 85% and 70% N of RFF plus the four PNSB strains were statistical equal to the treatment fertilized with only 100% N of RFF. In addition, the total N uptake increased along with the increase in the PNSB density in the soil, with a correlation coefficient of 0.6002 (Fig 2).

### The influence of N_2_-fixing purple non-sulfur bacteria on the growth and yield of canary melon

#### The growth of canary melon.

The height of canary melon among treatments changed significantly at 5%. In the treatment fertilized with only 100% N of RFF, the plant height were higher than those in the treatment fertilized with 70% N of RFF, with 256.4 compared to 246.0 cm, at 38 DAP. In the treatments fertilized with 100, 85 and 70% N of RFF plus mixed PNSB, the height was equivalent, i.e., 261.4, 260.3 and 260.1 cm, respectively, higher than those in the treatments fertilized with only chemical fertilizer at the same N fertilizer levels, except for the one fertilized with only 100% N of RFF. Application of only the four PNSB strains increased plant height, compared to the case of no bacteria and no chemical fertilizer, with 251.7 compared to 232.2 cm. Simultaneously, in the treatment supplemented with only the four PNSB strains, the plant height was equivalent to that in the treatments fertilized only with 85% and 70% N of RFF, 251.7 compared to 252.2 and 246.0 cm, respectively (Table 4).

**Table 4:** Influences of N_2_-fixing purple nonsulfur bacteria *R. palustris* on growth of canary melon cultivated on alluvial soil.

Treatment	Plant height	Stem diameter	Number of leaves	Position of fruit bearing leaf
	(cm)	(mm)	(leaves plant^-1^)	(leaf)
**100% N**	256.4 ± 1.7^ab^	8.44 ± 0.48^ab^	28.3 ± 0.7^bc^	11.1 ± 0.3
**85% N**	252.2 ± 2.0^bc^	8.32 ± 0.47^ab^	28.2 ± 0.6^c^	11.3 ± 2.8
**70% N**	246.0 ± 1.2^c^	8.26 ± 0.53^ab^	28.1 ± 0.3^c^	11.3 ± 0.4
**100% N + PNSB**	261.4 ± 6.5^a^	8.94 ± 0.65^a^	29.6 ± 0.2^ab^	11.1 ± 0.6
**85% N + PNSB**	260.3 ± 3.5^a^	8.68 ± 0.19^ab^	29.9 ± 1.0^a^	10.9 ± 0.4
**70% N + PNSB**	260.1 ± 5.2^a^	8.39 ± 0.44^ab^	29.1 ± 0.5^abc^	10.4 ± 1.1
**0% N + PNSB**	251.7 ± 0.9^bc^	7.91 ± 0.11^bc^	28.6 ± 0.7^bc^	11.0 ± 0.7
**0% N**	232.2 ± 1.6^d^	7.22 ± 0.43^c^	27.9 ± 0.8^c^	10.1 ± 0.2
Significant differences	*	*	*	ns
CV (%)	1.39	5.67	2.35	10.7

Numbers in each column with the same following letters are not significantly different from each other. ns: not significant difference; *: significant difference at 5% according to Duncan’s test; PNSB: Mixture of N_2_-fixing bacteria strains as *R. palustris* VNW64, VNS89, TLS06 and VNS02, N: Nitrogen.

The stem diameters at 38 DAP among treatments varied at 5% significance. However, the treatments fertilized with only chemical fertilizer had stem diameters ranging from 8.26 to 8.44 mm, different insignificantly from which the treatments fertilized with both chemical fertilizer and the four PNSB, 8.39–8.94 mm (Table 4). The treatment applied with only the four PNSB strains were statistically identical with the treatment with no fertilization.

At 38 DAP, the number of leaves per plant among treatments differed from each other at 5% significance. The modification of N fertilizer levels did not affect the number of leaves among treatments. In detail, in the treatment fertilized with only 100, 85, 70 and 0% N of RFF, the number of leaves was 28.3, 28.2, 28.1 and 27.9 leaves plant^-1^, while, in the treatments fertilized with 100, 85 70 and 0% N of RFF plus the four PNSB strains, the number of leaves were 29.6, 29.9, 29.1 and 27.9 leaves plant^-1^, respectively. The number of leaves per plant in the treatment supplemented with only the four PNSB strains was statistically equal to that in the treatment with no fertilization (Table 4).

The position of the fruit bearing leaf had no significant difference among treatments which neither applied only N fertilizer nor used both chemical fertilizer plus the four PNSB strains. The average position of fruit bearing leaf was 10.9 (Table 4).

#### The yield components of canary melon.

The fruit length valued differently among treatments at 5% significance. In detail, the reduction in the N fertilizer levels applied decreased the fruit length, with 13.6, 13.4 and 11.7 cm according to N fertilizer levels of 100, 85 and 70% N of RFF. In the treatment fertilized with 100% N of RFF plus the four PNSB strains, the fruit length peaked at 14.7 cm, the following longest was in the treatment fertilized with 85% N of RFF plus the four PNSB strains, 13.9 cm, and the shortest one was in the treatment fertilized with 70% N of RFF plus the four PNSB strains. In other words, the fruit length in treatments supplemented with the four PNSB strains was longer than those in the treatments supplemented with no bacteria at the same N fertilizer levels. In addition, in the treatment fertilized with 70% N of RFF plus the four PNSB strains, the fruit length was equivalent to that in the treatment fertilized with 100% N of RFF. Furthermore, the treatment supplemented with the four PNSB strains had a fruit length of 13.1 cm, which was longer than 11.2 cm in the treatment with no fertilization (Table 5).

**Table 5:** Influences of N_2_-fixing purple nonsulfur bacteria *R. palustris* on yield components of canary melon cultivated on alluvial soil.

Treatment	Fruit length	Fruit perimeter	Fruit weight
	(cm)	(cm)	(kg)
**100% N**	13.6 ± 0.2^c^	36.6 ± 0.8^ab^	0.800 ± 0.003^b^
**85% N**	13.4 ± 0.2 cd	35.3 ± 1.1^b^	0.766 ± 0.043^c^
**70% N**	11.7 ± 0.2^e^	35.3 ± 0.4^b^	0.747 ± 0.002 cd
**100% N + PNSB**	14.7 ± 0.1^a^	37.4 ± 0.2^a^	0.839 ± 0.013^a^
**85% N + PNSB**	13.9 ± 0.1^b^	36.8 ± 1.5^ab^	0.803 ± 0.013^b^
**70% N + PNSB**	13.5 ± 0.2 cd	36.1 ± 0.1^ab^	0.764 ± 0.010^c^
**0% N + PNSB**	13.1 ± 0.2^d^	34.9 ± 1.5^b^	0.726 ± 0.010^d^
**0% N**	11.2 ± 0.3^f^	31.7 ± 0.9^c^	0.664 ± 0.020^e^
Significant differences	*	*	*
CV (%)	1.40	2.76	2.54

Numbers in each column with the same following letters are not significantly different from each other. *: significant difference at 5% according to Duncan’s test; PNSB: Mixture of N_2_-fixing bacteria strains as *R. palustris* VNW64, VNS89, TLS06 and VNS02, N: Nitrogen.

The fruit perimeter varied at 5% significance among treatments. Although the fruit perimeter in the treatment supplemented with the four PNSB strains was bigger than that in the treatment with no fertilization, 34.9 compared to 31.7 cm, it did not change significantly between the combination of the four PNSB strains and the N fertilization and the use of only N fertilizer. To be more specific, from the N fertilizer levels of 70% to 100% N of RFF, the fruit perimeter was 35.3–36.6 cm in the absence of bacteria, while applying the four PNSB strains, the value was 36.1–37.4 cm (Table 5).

The fruit weight among treatments was different at 5% significance. In detail, the fruit weight dropped when the amount of N fertilizer levels was cut off, with 100%, 85% and 70% N of RFF correspondent to 794.6, 754.3 and 721.7 g of fruit weight. The treatments fertilized with the N fertilizer plus the four PNSB strains had the heaviest fruit at 100% N of RFF (840.4 g), the second heaviest at 85% N of RFF (800.2 g) and the least heavy at 70% N of RFF (743.8 g). Additionally, in the absence of chemical fertilizer, the fruit weight in the treatment supplemented with the four PNSB strains was 705.7 g and higher than that in the treatment with no PNSB supplementation, 640.2 g. Moreover, in the treatment fertilized with 85% N of RFF plus the four PNSB strains, the fruit weight was statistically equal to that in the treatment fertilized with 100% N of RFF (Table 5).

#### The yield of canary melon.

**Fig 3:**
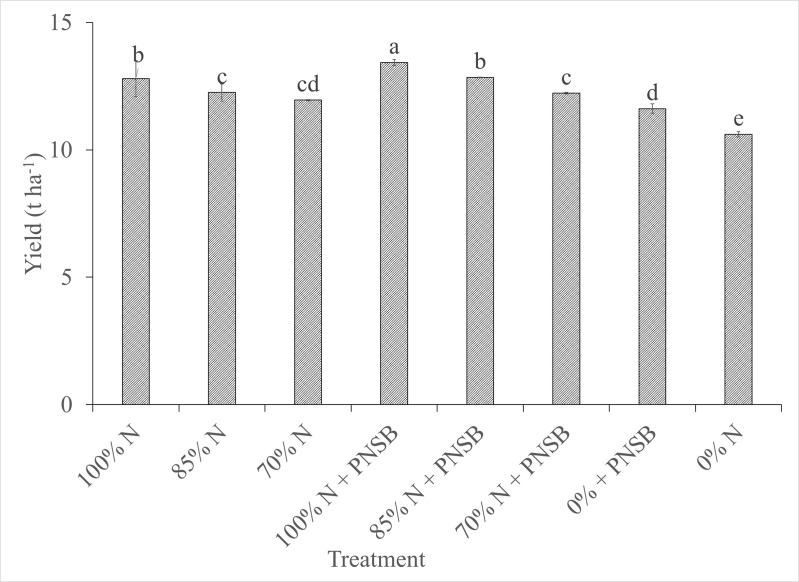
Influences of N_2_-fixing purple nonsulfur bacteria *R. palustris* on yield of canary melon cultivated on alluvial soil. PNSB: Mixture of N_2_-fixing bacteria strains as *R. palustris* VNW64, VNS89, TLS06 and VNS02, N: Nitrogen.

**Fig 4:**
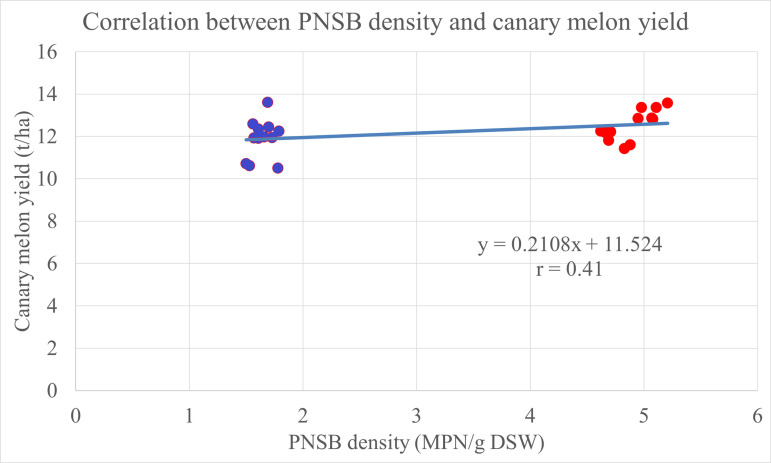
The correlation between the PNSB density in the soil and the yield of canary melon. Blue spots: treatments without PNSB; red spots: treatments with PNSB. MPN: Most Probable Number; DSW: dry soil weight.

The yield of canary melon changed at 5% significance among treatments. It was clearly seen that the reduction in the amount of N fertilizer used led to the decline in the yield of canary melon. In detail, the treatments fertilized with only N fertilizer levels at 0, 70, 85 and 100% N of RFF, the yield was 10.61 < 11.95 ~ 12.25 < 12.80 t ha^-1^, respectively. For the treatments fertilized with N fertilizer plus the four PNSB strains, at 100% N of RFF, the fruit yield was 13.43 t ha^-1^, 12.84 t ha^-1^ at 85% N of RFF and 12.22 t ha^-1^ at 70% N of RFF. The yield in these treatments was higher than those in the treatments using only N fertilizer at the same N fertilizer levels of 85 and 100% N of RFF. In the treatment supplemented with the four PNSB strains, the yield was 11.61 t ha^-1^ and higher than 10.61 t ha^-1^ in the treatment with no fertilization. Moreover, in the treatment fertilized with 85% N plus the four PNSB strains, the yield was 12.84 t ha^-1^, equivalent to that in the treatment fertilized with 100% N of RFF, 12.80 t ha^-1^. Notably, in the treatment supplemented with the four PNSB strains, the yield was 11.61 t ha^-1^, not significantly different from to 11.95 t ha^-1^ in the treatment fertilized with 70% N of RFF (Fig 3). Moreover, the canary melon yield proportionally correlated with the PNSB density in the soil, with a correlation coefficient of 0.41 (Fig 4).

### The influences of N_2_-fixing purple non-sulfur bacteria on the quality of canary melon

Shell thickness, flesh thickness, concentrations of nitrate, vitamin C, total acid and Brix index of canary melon among treatments had no significant differences. The mean values were 0.296 cm, 2.29 cm, 5.78 mg NO_3_^-^ kg^-1^, 19.4 mg 100 g^-1^, 0.172% and 15.4%, respectively (Table 6).

**Table 6:** Influences of N_2_-fixing purple nonsulfur bacteria *R. palustris* on quality of canary melon fruit cultivated on alluvial soil.

Treatment	Fruit hardness	Fruit shell colors			Shell thickness	Flesh thickness	NO_3_^-^	Vitamin C	Brix	Total acid	Storage time
		L*	a*	b*							
	(kgf cm^-2^)	–	–	–	(cm)	(cm)	(mg kg^-1^)	(mg 100 g^-1^)	(%)	(%)	(day)
**100% N**	1332.2 ± 141.4^bcd^	69.7 ± 0.1^b^	22.2 ± 0.3^abc^	73.8 ± 0.7^a^	0.267 ± 0.034	2.34 ± 0.15	5.88 ± 0.64	19.4 ± 1.8	16.0 ± 1.0	0.187 ± 0.046	17.7 ± 1.5^c^
**85% N**	1367.2 ± 206.7^bcd^	69.2 ± 0.7^b^	21.4 ± 1.8^bc^	74.2 ± 2.0^a^	0.289 ± 0.038	2.32 ± 0.19	5.41 ± 1.66	21.7 ± 1.1	15.0 ± 0.1	0.153 ± 0.006	17.3 ± 1.5^c^
**70% N**	1560.0 ± 215.5^abcd^	69.2 ± 1.6^b^	21.0 ± 1.4^c^	73.6 ± 0.8^ab^	0.324 ± 0.015	2.22 ± 0.15	5.54 ± 0.37	18.5 ± 0.9	14.7 ± 0.6	0.213 ± 0.031	16.7 ± 2.1^c^
**100% N + PNSB**	1709.7 ± 304.2^abc^	71.8 ± 0.2^a^	23.9 ± 1.1^a^	72.4 ± 1.1.^abc^	0.256 ± 0.020	2.43 ± 0.26	5.41 ± 1.91	20.1 ± 0.7	15.8 ± 1.0	0.160 ± 0.001	28.0 ± 2.6^a^
**85% N + PNSB**	1730.0 ± 0.1^ab^	72.7 ± 0.4^a^	24.0 ± 0.3^a^	71.8 ± 1.1.^bc^	0.317 ± 0.034	2.30 ± 0.26	5.29 ± 0.55	20.4 ± 0.9	15.7 ± 1.2	0.160 ± 0.010	26.0 ± 2.0^ab^
**70% N + PNSB**	1952.1 ± 41.3^a^	72.7 ± 1.0^a^	23.5 ± 1.2^ab^	71.5 ± 0.4^c^	0.278 ± 0.019	2.41 ± 0.19	5.95 ± 0.54	18.3 ± 2.1	14.8 ± 0.8	0.187 ± 0.023	27.0 ± 2.0^a^
**0% N + PNSB**	1295.3 ± 120.0 cd	71.4 ± 1.2^a^	22.2 ± 1.0^abc^	71.6 ± 0.7^c^	0.322 ± 0.051	2.16 ± 0.25	6.53 ± 0.95	18.8 ± 0.2	15.3 ± 0.6	0.160 ± 0.030	22.7 ± 1.5^b^
**0% N**	1257.2 ± 352.8^d^	69.1 ± 0.4^b^	21.4 ± 1.7^bc^	73.8 ± 0.9^a^	0.311 ± 0.042	2.11 ± 0.36	6.25 ± 0.26	17.6 ± 3.1	15.8 ± 0.3	0.157 ± 0.012	14.3 ± 2.1^c^
Significant differences	*	*	*	*	ns	ns	ns	ns	ns	Ns	*
CV (%)	14.4	1.24	5.38	1.35	10.7	9.38	18.8	8.30	5.34	18.4	9.53

Numbers in each column with the same following letters are not significantly different from each other. ns: not significant difference; *: significant difference at 5% according to Duncan’s test; PNSB: Mixture of N_2_-fixing bacteria strains as *R. palustris* VNW64, VNS89, TLS06 and VNS02, N: Nitrogen.

Fruit hardness, index of L*, a* and b*, and storage time differed significantly at 5% according to treatments. In detail, the fruit hardness fluctuated from 1332.2 to 1560.0 kgf cm^-2^ in the treatments fertilized with only chemical fertilizer and from 1709.7 to 1952.1 kgf cm^-2^ in the treatments with the combination of N fertilizer levels and the four PNSB strains. Additionally, the average L* index in the treatments fertilized with both N fertilizer levels and the four PNSB strains was 72.4 and higher than that in the treatments fertilized with only N fertilizer, with 69.4. Moreover, in the case without chemical fertilizer, the treatment supplemented with the four PNSB strains had an L* index of 71.4, higher than 69.1 in the treatment with no bacteria. Furthermore, the a* index in the treatments fertilized with the combination of the N fertilizer levels and the four PNSB strains was higher than those at the same N fertilizer levels but no bacteria, except for the treatment fertilized with 100% N of RFF. The b* index in the treatments fertilized with only N fertilizer or in the ones fertilized with both N fertilizer levels and the four PNSB strains shared the same trend as the index of a*. In the treatment fertilized with 0% N of RFF plus no bacteria, the index of b* was 73.8, higher than that in the treatment supplemented with the four PNSB strains, 71.6. The storage time of the canary melon in the treatments fertilized with the N fertilizer plus the four PNSB strains was roughly 26.0–28.0 days, longer than 17.3–17.7 days in the treatments fertilized with only N fertilizer at the same levels. While there was no N fertilizer applied, the treatment supplemented with the four PNSB strains had the storage time of 22.7 days, longer than 14.3 days in the treatment with no bacteria (Table 6).

### Discussion

Soil characteristics of pH_H2O_ and pH_KCl_ were low, approximately at 4.39 and 3.84 (Table 1), which can hinder nutrient availability for plants. According to Cerozi and Fitzsimmons [24], soil pH significantly influences nutrient solubility and uptake. An increase in pH toward neutral conditions enhances the efficiency of nitrogen (N) and phosphorus (P) utilization by plants [25]. The result in Table 2 showed that the treatments supplemented with the four PNSB strains raised the values of pH_H2O_, in comparison to the treatments with no bacteria, 5.00–5.46 compared to 4.38–5.15, respectively (Table 2). This pH increase is likely attributable to bacterial production of compounds such as ALA and EPS, which can neutralize soil acidity and buffer against further acidifications [14,15,26]. Therefore, enhanced soil pH was associated with increased NH_4_^+^ concentrations in PNSB-treated soils. For further evaluation, the treatments fertilized with only N fertilizer had the available N concentration at 12.0–16.1 mg NH_4_^+^ kg^-1^, while the treatments fertilized with the N fertilizer levels and the four PNSB strains had the result of 14.3–18.9 mg NH_4_^+^ kg^-1^ (Table 2). In the absence of chemical fertilizer, concentration of NH_4_^+^ was recorded to be 6.87 mg NH_4_^+^ kg^-1^ (Table 2), equivalent to the amount the soil at the beginning the crop, 7.28 mg NH_4_^+^ kg^-1^ (Table 1). These amounts of available N were lower than that in the treatment supplemented with only the four PNSB strains, 11.7 mg NH_4_^+^ kg^-1^ (Table 2). These findings support the N₂-fixing capacity of *R. palustris* VNW64, VNS89, TLS06, and VNS02, consistent with previous reports demonstrating their activity in acidic and neutral soils [14,27]. Thus, these strains can fix N_2_ in various types of soil, including acidic sulfate and alluvial soils. Simultaneously, the strains of *R. palustris* VNW64, VNS89, TLS06 and VNS02 have P-solubilizing function as well [15], from which, in the depth of 0–20 cm, in the treatments supplemented with the four PNSB strains, the concentration of soluble P was higher and the contents of Al-P, Fe-P and Ca-P was lower, compared to those in the treatments without bacteria supplementation (Table 2). This could be interpreted by the production of acid phosphatase by *Rhodopseudomonas* spp. as a P-solubilizing mechanism [15]. As the result, the application of the bacterial strains participated in freeing a mass amount of P from its insoluble forms in initial soil (Table 1). Comparable results were observed in a study by Artyszak and Gozdowski [28] in beetroot supplied with *Azotobacter chroococcum*, *A. brasilense* and *Bacillus megaterium* combined with a reduction by 30% of RFF, leading to a dominance in soil NO_3_^-^, NH_4_^+^, compared to the control treatment where only chemical fertilizer is applied. Moreover, on canary melon, the current study successfully replaced the N fertilization by strains of PNSB, while the P solubilization by the PNSB strains have been investigated by Xuan et al. [29] Although the bacterial strains of *R. palustris* VNW64, VNS89, TLS06 and VNS02 were not determined their ability to solubilize potassium forms, the treatments supplemented the four PNSB strains, the potassium concentration was 0.456 meq K^+^ 100 g^-1^ and higher than that in the treatments with no bacteria, 0.358 meq K^+^ 100 g^-1^ (Table 2). However, the strain of *R. palustris* G5 has been proved its capability of potassium solubilization [30]. This took part in explaining why potassium concentration in the treatment supplemented with the four PNSB strains was higher than that in the treatment with no bacteria plus either N fertilizer or no fertilization (Table 2).

The PNSB strains did fix N_2_ in the atmosphere to NH_4_^+^ in soil, which played as the N supply to plants (Table 2). This is in accordance with a study by Maeda [31], where the PNSB strains are capable of fixing N. Therefore, strains of *R. palustris* help to improve the availability of macronutrients, including N, P and K in soil [32]. The N concentration in stem, leaves in the treatments supplemented with the four PNSB strains was higher than those in the treatments with no bacteria at the same N fertilizer levels. In detail, the N concentration in stem, leaves of canary melon in the treatments fertilized with 70, 85 and 100% N of RFF plus the four PNSB strains was 2.52, 2.61 and 2.62% N, which was higher than those in the treatments fertilized with only N fertilizer, 2.27, 2.34 and 2.44% N, respectively (Table 3). Thus, the application of the PNSB enhanced biomass in dry stem, leaves and dry fruits of the canary melon. In the same, the PNSB also raised the N uptake in the treatments fertilized with chemical fertilizer plus the PNSB, compared to that in the treatments without bacteria at the same N fertilizer levels (Table 3). The total N uptake in the treatments fertilized with only N fertilizer levels of 70, 85 and 100% N of RFF was 23.4, 25.6 and 28.6 kg N ha^-1^, respectively. Nevertheless, in the treatments fertilized with 70, 85 and 100% N of RFF plus the four PNSB strains, total N uptake was higher than those in the treatments fertilized with only N fertilizer levels, with 27.7, 31.7 and 33.9 kg N ha^-1^, respectively (Table 3). Moreover, the supplementation of *R. palustris* PS3 increased N uptake in Chinese cabbage. This could be explained that the application of the bacteria stimulated the production of endogenous auxin in mature leaves in order to increase leaf cells during their developing stage [12]. Therefore, the supplementation of *R. palustris* increased the N concentration in leaves (Table 3). Simultaneously, the N fixation in soil also promotes the growth of plants. As the result, the application of *R. palustris* enhanced N uptake of canary melon (Table 3). This result was consistent with previous studies, in which the supplementation of *R. palustris* raised the N uptake of rice cultivated on acid sulfate soil [15,26,33]. Ultimately, to summarize the correlation, according to Figs 1, 2, and 4, the PNSB density in the soil proportionally correlated with the NH_4_^+^ concentration in the soil, the total N uptake in the plant, and the canary melon yield, with r = 0.4553, 0.6002, and 0.41, respectively. This indicated that the PNSB supplementation increased the N availability in the soil, leading to better total N uptake and eventually the crop yield. This pointed out that the bacterial strains of *R. palustris* VNW64, VNS89, TLS06 and VNS02 were effective not only for rice in submerged condition, but also for terrestrial plants, this is due to their ability to live under various conditions, including photoautotroph, photoheterotroph, chemoautotroph and chemoheterotroph [13,19,34].

Fertilizing with 100% N of RFF increased plant height by 10.4 and 24.2 cm, compared to fertilizing with 70% N of RFF and with no chemical fertilizer (Table 4). This showed that canary melon responded to N fertilizer. The result was in accordance with a study by Aluko [35], where canary melon using a fertilizer of NPK 15-15-15 with 333 kg ha^-1^, plant height is higher than that in the control treatment with no fertilizer, with 115.7 cm compared to 86.1 cm, respectively. Furthermore, in the treatments fertilized with both N fertilizer levels and the four PNSB strains, the fruit height rose at 13.5, 13.9 and 14.7 cm, compared to those in the treatments fertilized with only N fertilizer levels, whose result was 11.7, 13.4 and 13.6 cm, following the increasing N fertilizer levels of 70, 85 and 100% of RFF (Table 5). As reported by Zahedyan et al. [36], fertilizing 120 kg ha^-1^ of chemical fertilizer and Nitroxin with 100% demand of irrigated water outputs the heaviest fruit weight (1.84 kg) and the longest fruit (20.71 cm). According to Maeda [31], inoculating PNSB to rice seeds shows efficiency in increasing the N uptake, growth and yield. The strains of *R. palustris* have characteristics of N_2-_fixing, and stimulating growth and yield of rice [15,27]. The result of this study also showed a 15% decrease in the N fertilizer while applying the four PNSB strains, but growth and yield of the canary melon also remained equal to those in the treatment fertilized with only 100% N of RFF. This was because these strains were able to fix N and secrete plant growth promoting compounds (Table 4). This result was in accordance with a conclusion of Khuong et al. [15] who claim that the bacterial strains of *R. palustris* VNW64, VNS89, TLS06 and VNS02 reduced 25% N of RFF for rice cultivation. Therefore, the application of the bacterial mixture promoted plant growth which consisted of plant height, number of leaves and stem diameter, as well as the yield components, including fruit length, fruit perimeter and fruit weight (Tables 4 and 5).

Góes et al. [37] conclude that fertilizer application puts influences on the hardness of fruit flesh, total soluble solid compounds concentration and total soluble carbohydrate in the melon. Yam et al. [38] report that the amount of N in hydroponic solution increases the hardness of cantaloupe. However, the hardness was not different statistically between N fertilizer levels (Table 6). According to Piñero et al. [39], increasing N fertilizer levels leads to the rise in index of L* and a*, while Ferrante et al. [40] report increase in L* and b* index of melon. In this study, index of L* and a* had the rising trend, while a downtrend was reported in b* index (Table 6). This meant that the application of the four PNSB strains gave melons with better colors. Moreover, qualitative parameters, including concentrations of nitrate, vitamin C and total acid and Brix index, remained as compared to the treatments without bacteria (Table 6). Although evaluating the storage time was not the main purpose of this study, this initial step showed that the application of the four PNSB strains elongated the time of preserve. To be more specific, the treatments fertilized with the four PNSB strains had the storage time of approximately 27.0 days, while in the treatments with no bacteria, the preservation was roughly 17.2 day; and 22.7 and 14.3 days in the case with no chemical fertilizer (Table 6). Although, the pathogenic fungi were not identified, based on the longer storage time, the mixture of strains of *R. palustris* VNW64, VNS89, TLS06 and VNS02 might have antagonistic activity against the fungi causing rotten fruits. Based on a study by Nookongbut et al. [41], the strains of *R. palustris* KTSSR54 antagonizes the pathogenic fungi, including *Bipolaris oryzae* NPT0508, *Curvularia lunata* SPB0627 and *Magnaporthe oryzae* PTRC63 by producing EPS compounds. The strains of *R. palustris* VNW64, VNS89, TLS06 and VNS02 in this study are able to secrete EPS [17], thus, they are potential in preventing some diseases.

## Conclusions

The supplementation of four strains of *R. palustris* VNW64, VNS89, TLS06 and VNS02 ameliorated the soil fertility, growth, yield and storage time of canary melon fruit. The application of mixed PNSB strains of *R. palustris* VNW64, VNS89, TLS06 and VNS02 enhanced pH by 0.25–1.08, soil NH_4_^+^ concentration by 2.30–4.83 mg NH_4_^+^ kg^-1^, total N uptake in plant by 4.30–12.1 kg N ha^-1^, plant height by 5.0–19.5 cm and fruit yield by 0.27–1.00 t ha^-1^, compared to no bacteria applied at the same N fertilizer levels. While applying 85% N of RFF plus the four PNSB strains of *Rhodopseudomonas* spp., NH_4_^+^ concentration, total N uptake, plant height and yield was equivalent to those in the treatment fertilized with only 100% N of RFF. Notably, the treatment combining 85% of the recommended nitrogen fertilizer with the PNSB mixture achieved plant growth and fruit yield equivalent to the full 100% RFF treatment, indicating that synthetic N inputs can be reduced by up to 15% without compromising productivity. Furthermore, even a 30% reduction in N fertilizer, when paired with PNSB, maintained equivalent N uptake levels. These findings highlight the potential of *R. palustris* as a biofertilizer to reduce reliance on chemical fertilizers while sustaining soil health and crop yield. This strategy contributes to more sustainable and environmentally friendly horticultural practices.

## Supporting information

S1 Table Influences of N_2_-fixing purple nonsulfur bacteria *R. palustris* on alluvial soil fertility in depth of 20–40 cm. (DOCX)
