# Supplementary material for: Effects of adding N2-fixing Rhodopseudomonas palustris to stimulate the growth and yield of canary melon (Cucumis melo L.)
Source: PLoS One. 2025 Aug 7;20(8):e0329938. doi: 10.1371/journal.pone.0329938 (PMC12331036; doi:10.1371/journal.pone.0329938)
Supplement: S1 Table — (DOCX) [file pone.0329938.s001.docx]

**S1 Table. Influences of N_2_-fixing purple nonsulfur bacteria R. palustris on alluvial soil fertility in depth of 20-40 cm**

| **Treatment** | **pH_H2O_** | **pH_KCl_** | **EC** | **Total N** | **NH_4_^+^** | **NO_3_^-^** | **Total P** | **Soluble P** | **Insoluble P forms** | | | **CEC** | **Cations** | | | |
| --- | --- | --- | --- | --- | --- | --- | --- | --- | --- | --- | --- | --- | --- | --- | --- | --- |
|  |  |  |  |  |  |  |  |  | **Al-P** | **Fe-P** | **Ca-P** |  | **K^+^** | **Na^+^** | **Ca^2+^** | **Mg^2+^** |
|  | **-** | **-** | **(mS cm^-1^)** | **(%)** | **(mg kg^-1^)** | | **(%)** | **(mg kg^-1^)** | | | | **(meq 100 g^-1^)** | | | | |
| **100% N** | 3.91 | 3.20 | 0.850^a^ | 0.107 | 15.6^abc^ | 21.1 | 0.060 | 72.6 | 61.2^ab^ | 260.7 | 56.0 | 13.4 | 0.330 | 0.472^a^ | 5.90^ab^ | 1.89^b^ |
| **85% N** | 3.85 | 3.31 | 0.653^bc^ | 0.122 | 12.1^cd^ | 22.2 | 0.048 | 78.2 | 56.3^bc^ | 244.4 | 46.7 | 13.6 | 0.330 | 0.470^a^ | 4.74^b^ | 1.88^b^ |
| **70% N** | 3.91 | 3.38 | 0.807^ab^ | 0.132 | 14.2^bc^ | 28.8 | 0.061 | 77.1 | 68.9^a^ | 272.1 | 54.2 | 14.6 | 0.366 | 0.311^bc^ | 6.50^a^ | 2.08^ab^ |
| **100% N + PNSB** | 3.98 | 3.30 | 0.703^ab^ | 0.128 | 18.0^ab^ | 24.5 | 0.056 | 79.0 | 59.9^ab^ | 270.6 | 51.6 | 14.4 | 0.341 | 0.272^bcd^ | 6.18^ab^ | 2.09^ab^ |
| **85% N + PNSB** | 3.93 | 3.36 | 0.470^d^ | 0.110 | 18.8^a^ | 21.3 | 0.051 | 67.7 | 59.9^ab^ | 233.6 | 52.2 | 13.9 | 0.330 | 0.202^cd^ | 4.70^b^ | 1.96^ab^ |
| **70% N + PNSB** | 4.01 | 3.40 | 0.667^bc^ | 0.117 | 14.4^bc^ | 24.6 | 0.053 | 79.5 | 59.1a^b^ | 248.8 | 47.2 | 13.7 | 0.354 | 0.171^d^ | 5.98^ab^ | 2.00^ab^ |
| **0% N + PNSB** | 4.41 | 4.04 | 0.407^d^ | 0.105 | 12.4^cd^ | 20.5 | 0.057 | 76.6 | 45.2^d^ | 263.1 | 48.0 | 14.4 | 0.326 | 0.239^bcd^ | 6.34^ab^ | 2.20^a^ |
| **0% N** | 4.23 | 3.37 | 0.540^cd^ | 0.113 | 9.23^d^ | 24.1 | 0.051 | 77.7 | 48.6^cd^ | 230.9 | 48.5 | 14.1 | 0.312 | 0.319^b^ | 6.34^ab^ | 2.00^ab^ |
| Significant differences | ns | ns | * | ns | * | ns | ns | ns | * | ns | ns | ns | ns | * | ns | ns |
| CV (%) | 6.64 | 10.6 | 13.1 | 13.7 | 15.8 | 12.8 | 13.3 | 16.3 | 9.68 | 11.1 | 9.64 | 13.3 | 13.3 | 19.4 | 13.6 | 7.54 |

*Numbers in each column with the same following letters are not significantly different from each other. ns: not significant difference; *: significant difference at 5% according to Duncan’s test; PNSB: Mixture of N_2_-fixing bacteria strains as R. palustris* VNW64, VNS89, TLS06 *and* VNS02*, N: Nitrogen, P: Phosphorus; EC: Electrical conductivity, CEC: Cation exchangeable capacity.*
